# First-line exome sequencing in Palestinian and Israeli Arabs with neurological disorders is efficient and facilitates disease gene discovery

**DOI:** 10.1038/s41431-020-0609-9

**Published:** 2020-03-25

**Authors:** Holger Hengel, Rebecca Buchert, Marc Sturm, Tobias B. Haack, Yvonne Schelling, Muhammad Mahajnah, Rajech Sharkia, Abdussalam Azem, Ghassan Balousha, Zaid Ghanem, Mohammed Falana, Osama Balousha, Suhail Ayesh, Reinhard Keimer, Werner Deigendesch, Jimmy Zaidan, Hiyam Marzouqa, Peter Bauer, Ludger Schöls

**Affiliations:** 1 Department of Neurology and Hertie-Institute for Clinical Brain Research, University of Tübingen, Tübingen, Germany; 2 German Center of Neurodegenerative Diseases (DZNE), Tübingen, Germany; 3 Institute of Medical Genetics and Applied Genomics, University of Tübingen, Tübingen, Germany; 4 The Ruth and Bruce Rappaport Faculty of Medicine, Technion, Haifa, Israel; 5 Child Neurology and Development Center, Hillel-Yaffe Medical Center, Hadera, Israel; 6 Unit of Nature Science, Beit-Berl Academic College, Beit-Berl, Israel; 7 Unit of Human Biology and Genetics, The Triangle Regional Research and Development Center, Kafr Qari, Israel; 8 Department of Biochemistry and Molecular Biology, Faculty of Life Sciences, Tel-Aviv University, Tel-Aviv, Israel; 9 Department of Pathology and Histology, Al-Quds University, Eastern Jerusalem, Palestine; 10 Palestine Medical Complex, Ramallah, Palestine; 11 Faculty of Medicine, Al-Quds University, Eastern Jerusalem, Palestine; 12 Molecular Genetics Laboratory, Al-Makassed Islamic Charitable Hospital, Jerusalem, Israel; 13 Caritas Baby Hospital Bethlehem, Bethlehem, Palestine; 14 Centogene AG (Rostock), Tübingen, Germany

**Keywords:** Genetics research, Neurological disorders, Genetic testing

## Abstract

A high rate of consanguinity leads to a high prevalence of autosomal recessive disorders in inbred populations. One example of inbred populations is the Arab communities in Israel and the Palestinian Authority. In the Palestinian Authority in particular, due to limited access to specialized medical care, most patients do not receive a genetic diagnosis and can therefore neither receive genetic counseling nor possibly specific treatment. We used whole-exome sequencing as a first-line diagnostic tool in 83 Palestinian and Israeli Arab families with suspected neurogenetic disorders and were able to establish a probable genetic diagnosis in 51% of the families (42 families). Pathogenic, likely pathogenic or highly suggestive candidate variants were found in the following genes extending and refining the mutational and phenotypic spectrum of these rare disorders: *ACO2*, *ADAT3*, *ALS2*, *AMPD2*, *APTX*, *B4GALNT1*, *CAPN1*, *CLCN1*, *CNTNAP1*, *DNAJC6*, *GAMT*, *GPT2*, *KCNQ2*, *KIF11*, *LCA5*, *MCOLN1*, *MECP2*, *MFN2*, *MTMR2*, *NT5C2*, *NTRK1*, *PEX1*, *POLR3A*, *PRICKLE1*, *PRKN*, *PRX*, *SCAPER*, *SEPSECS*, *SGCG*, *SLC25A15*, *SPG11*, *SYNJ1*, *TMCO1*, and *TSEN54*. Further, this cohort has proven to be ideal for prioritization of new disease genes. Two separately published candidate genes (WWOX and PAX7) were identified in this study. Analyzing the runs of homozygosity (ROHs) derived from the Exome sequencing data as a marker for the rate of inbreeding, revealed significantly longer ROHs in the included families compared with a German control cohort. The total length of ROHs correlated with the detection rate of recessive disease-causing variants. Identification of the disease-causing gene led to new therapeutic options in four families.

## Introduction

Consanguinity is a deeply rooted cultural trait in Middle Eastern societies, especially in the Arab rural populations due to socio-cultural factors like maintenance of the family structure, property, or ease of marital arrangements [1]. Despite the fact that this type of marriages is discouraged by the major religions, recent studies estimated the prevalence of consanguineous marriages among the Palestinian Arab and Israeli Arab population to 44.3% and 25.9%, respectively, representing some of the highest rates in the world [2–5]. This high inbreeding rate leads to a high prevalence of autosomal recessive disorders. In first cousin relations, the risk of significant birth defects is increased up to 2.5 times as compared with the general population [3]. Especially in rural Palestinian areas these patients do not have access to advanced medical diagnostics and can often not be assessed by trained specialists. Therefore, most of the time a genetic diagnosis is not established.

In this study, we performed first-line whole-exome sequencing in 83 Arab families with suspected neurogenetic disorders due to at least two similar affected patients to identify the genetic cause of disease. Starting from only minimal clinical information, WES identified potentially disease-causing variants that were confirmed in a second step by targeted reverse phenotyping. Through this, 37 families received a definite genetic diagnosis and 5 families a likely diagnosis with novel candidate variants, leading to a high diagnostic yield of ~51%. Moreover, a specific therapy was made possible in four families due to WES.

## Methods

Families were identified and enrolled in Israel or the Palestinian territories by cooperating physicians from 2012 to 2017. The inclusion criteria were defined as follows: (1) patients had to present with so far unexplained neurological symptoms and (2) at least two family members, including the index patient, had to suffer from similar symptoms. Initial phenotyping was often performed by medical staff who were not trained as neurologists and was thus mostly limited to broad categories such as “movement disorder,” “intellectual disability,” and “epilepsy.”

Written informed consent was obtained from the patients or the parents of the underage patients for diagnostic procedures and next-generation sequencing. The study has been approved by the local Institutional Review Board (vote 180/2010BO1).

### Genetics

Patients were screened for exonic variants using a whole-exome enrichment approach (SureSelectXT Human All Exon V5 or SureSelectXT Human All Exon V6; Agilent, Santa Clara CA). Sequencing was performed on a HiSeq2500 (200 cycle chemistry) or NextSeq500 (300 cycle chemistry) platform (Illumina, San Diego, CA) in paired-end mode according to the manufacturers’ protocol.

Data analysis was performed using the megSAP [6] pipeline. The pipeline uses BWA-MEM [7] for alignment, freebayes [8] for variant calling, and Ensembl VEP [9] for variant annotation. Variants were first checked for pathogenic variants known to be associated with neurological disorders using the HGMD [10] database. If no known disease-causing variant was identified, variants were next filtered for rare variants (gnomAD [11] minor allele frequency <0.1%), considering both recessive and dominant inheritance, and prioritized according to gene function, conservation (pyhloP [12], GERP++ [13]) and in silico prediction scores (CADD [12], SIFT [14], PolyPhen2 [15]). If no clear candidate variant could be identified, a second exome was sequenced from another affected family member to reduce the number of potential variants. A total of 102 exomes were sequenced. In addition, copy number variants were determined from WES data using CnvHunter [16], a tool which compares the depth of coverage for each exon to a collective of reference samples to determine outlier exons. Runs of homozygosity (ROHs) were determined using RohHunter [16], which detects homozygous regions that are too long to occur by chance based on detected SNP genotypes and the allele frequency of the SNPs in public databases. Sanger sequencing was used to confirm the identified variants and test the segregation in all available family members. For nonsegregating variants, the possibility of two independent genetic disorders was taken into account. Variants have been classified as pathogenic or likely pathogenic if they fulfilled the respective ACMG criteria [17]. The respective conditions under which the variants are causing disease (dominant, recessive, X-linked) are specified for each pathogenic/likely pathogenic variant in Table 1 and Table S1. Patients presenting with cerebellar ataxia were additionally screened for repeat expansions in SCA 1, 2, 3, 6, 7, 17 and Friedreich’s ataxia. Patients with HSP phenotypes underwent MLPA for SPG4. All WES data from unsolved cases were reannotated and reanalyzed shortly before submission of the paper.

**Table 1:** Overview of identified pathogenic and likely pathogenic variants and observed phenotypes.

Gene	MIM	Affected/unaffected	FamilyID	Phenotype	Variant	Zygosity	Novel/known, classification, inheritance
*ACO2*	614559	2/3	TR20* [34]	Intellectual disability, truncal hypotonia, strabismus	NM_001098.3:c.336C>G: p.(Ser112Arg)	hom	Known variant [35], pathogenic, AR
*ADAT3*	615286	2/4	BE05	Intellectual disability, truncal hypotonia, strabismus	NM_001329533.1:c.382G>A: p.(Val128Met)	hom	Known variant [36], pathogenic, AR
*ALS2*	205100	2/6	BE15	Tetraparesis with affection of upper and lower motor neuron	NM_001135745.1:c.601C>T: p.(Arg201*)	hom	Novel variant, likely pathogenic, AR
*AMPD2*	615809	3/2	MA07	Severe psychomotor retardation, pontocerebellar hypoplasia, microcephaly	NM_001257360.2:c.495del: p.(Gln166Argfs*)21	hom	Known variant [37], pathogenic, AR
*APTX*	208920	1/3	TR03A	Cerebellar ataxia, oculomotor apraxia	NM_175073.2:c.837G>A: p.(Trp279*)	hom	Known variant [38], pathogenic, AR
*B4GALNT1*	609195	3/6	AQ35	Spastic paraparesis, intellectual disability	NM_001276469.1:c.263dup: p.(Leu89Profs*13)	hom	Novel variant, likely pathogenic, AR
*CAPN1*	616907	3/3	AQ28	Spastic paraparesis	NM_005186.3:c.1605+5G>A: p.(?)	hom	Known variant [39], pathogenic, AR
*CLCN1*	255700	1/8	AQ47	Weakness, myotonia, muscle pain, muscle hypertrophy in calves	NM_000083.3:c.1012C>T: p.(Arg338*)	hom	Known variant [40], pathogenic, AR
*CNTNAP1*	618186	3/11	TR30* [26]	Intellectual disability, epilepsy, arthrogryposis	NM_003632.3:c.2015G>A: p.(Trp672*)	hom	New variant* [26], pathogenic, AR
*DNAJC6*	615528	2/4	AQ01	Early-onset parkinsonism and dystonia	NM_001256864.2:c.801–2A>G: p.(?)	hom	Known variant [41], pathogenic, AR
*GAMT*	612736	2/5; 2/6; 3/4	AQ39; AQ40; TR28	Intellectual disability, movement disorder with myoclonic jerks	NM_138924.2:c.491del: p.(Gly164Alafs*14)	hom	Known variant [42], pathogenic, AR
*GPT2*	616281	3/4, 2/3	BE01*; BE02* [17]	Intellectual disability, spastic paraparesis, epilepsy, microcephaly	NM_133443.4:c.70C>T: p.(Gln24*)	hom	Novel variant* [17], pathogenic, AR
*LCA5*	604537	2/6	TR19A	Night blindness	NM_181714.3:c.1062_1068del: p.(Tyr354*)	hom	Known variant [43], pathogenic, AR
*MTMR2*	601382	2/6, 2/9	AQ18, AQ19	Severe peripheral neuropathy, facial weakness, hoarseness, tetraplegia, muscle wasting	NM_016156.5:c.766_767del,p.(Lys256Glufs*20)	hom	Novel variant, likely pathogenic, AR
*NT5C2*	613162	1/2	TR13	Spastic paraparesis, intellectual disability	NM_012229.4:c.430C>T: p.(Arg144*)	hom	Novel variant, likely pathogenic, AR
*NTRK1*	256800	1/3	AQ32	Sensory neuropathy, anhidrosis, insensitivity to pain	NM_002529.3:c.2170G>A: p.(Gly724Ser)	hom	Novel variant, likely pathogenic, AR
*PAX7*	n/a	1/4	BE07* [25]	Muscular dystrophy	NM_013945.2:c.220C>T: p.(Arg74*)	hom	Novel gene* [25], likely pathogenic, AR
*PEX1*	214100	2/6	BE08	Epilepsy, intellectual disability, schizencephaly	NM_001282677.1:c.403C>T: p.(Arg135*)	hom	Novel variant, likely pathogenic, AR
*POLR3A*	607694	2/3	TR07* [27]	Akinesia, rigidity, ataxia, pyramidal tract affection, intellectual disability	NC_000010.10(NM_007055.3):c.1771-7C>G: p.(?)	hom	New variant* [27], pathogenic, AR
*PRICKLE1*	612437	4/11, 2/3	TR01; TR36	Cerebellar ataxia, epilepsy	NM_001144881.1:c.311G>A: p.(Arg104Gln)	hom	Known variant [44], pathogenic, AR
*PRKN*	600116	4/5	TR10	Parkinsonism	NM_004562.3:c.101delA: p.(Gln34Argfs*10)	hom	Known variant [45], pathogenic, AR
*PRX*	614895	2/4	TR29	Peripheral neuropathy	NC_000019.9(NM_181882.2):c.27+1G>T: p.(?)	hom	Novel variant, likely pathogenic, AR
*SCAPER*	618195	3/3	TR34	Intellectual disability, retinitis pigmentosa, cataracts	NC_000015.10(NM_020843.2):c.2023-2A>G: p.(?)	hom	Known variant [28], pathogenic, AR
*SGCG*	253700	2/8	AQ36	Limb-girdle muscular dystrophy	NM_000231.2:c.525delT: p.(Phe174Leufs*20)	hom	Known variant [46], pathogenic, AR
*SGSH*	252900	2/2	TR02* [47]	Intellectual disability, autistic behavior, spasticity	NM_000199.5:c.416C>T: p.(Thr139Met)	hom	Known variant* [47, 48], pathogenic, AR
*SlC25A15*	238970	2/4	AQ16	Spastic paraparesis, cerebellar ataxia, mild polyneuropathy	NM_014252.4:c.446delG: p.(Ser149Thrfs*45)	hom	Variant in Clinvar as pathogenic, likely pathogenic, AR
*SPG11*	604360	3/6	AQ54	Spastic paraparesis, intellectual disability	NM_025137.4:c.4307_4308del: p.(Gln1436Argfs*7)	hom	PMID: 18079167 [49], pathogenic, AR
*TMCO1*	213980	2/4	TR24*	Intellectual disability, epilepsy, corpus callosum dysgenesis	NM_019026.4:c.616C>T: p.(Arg206*)	hom	Novel variant, likely pathogenic, AR
*WWOX*	616211	2/2	TR08* [24]	Cerebellar ataxia, intellectual disability	NM_016373.4:c.1114G>C: p.(Gly372Arg)	hom	Novel gene* [24], likely pathogenic, AR
*KCNQ2*	613720	2/3	TR17	Early infantile epileptic encephalopathy	NM_172109.2:c.913_915del: p.(Phe305del)	het	Novel variant, likely pathogenic, AD
*KIF11*	152950	5/0	TR35	Intellectual disability, microcephaly, chorioretinopathy	NM_004523.4:c.381G>A: p.(Trp127*)	het	Novel variant, likely pathogenic, AD
*MECP2*	300055	1/5	BE12	Mental retardation, epilepsy	NM_004992.3:c.925C>T: p.(Arg309Trp)	hemi	Known variant [50], pathogenic, XLR

Families that have been published separately are marked with asterisk (*) and the respective reference is given. Detailed annotations can be found in Table S1.

*AR* autosomal recessive, *AD* autosomal dominant, *MIM* mendelian inheritance in men, *XLR* X-linked recessive.

### Statistical analysis

**Fig. 1:**
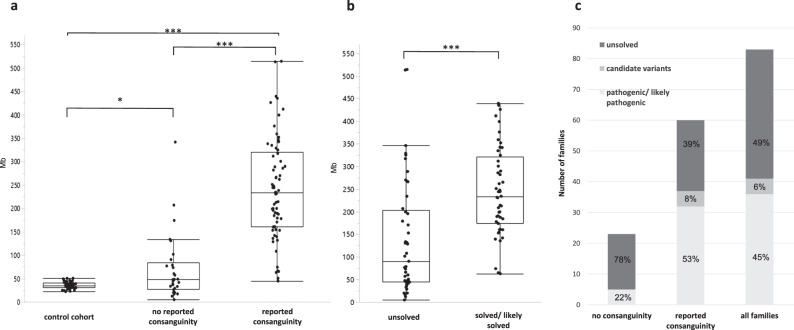
Overview of the cohort. **a** Runs of homozygosity (ROHs) in a German control cohort, patients with no reported consanguinity and patients with reported consanguinity. Every dot represents the ROHs in one exome. The ROHs are significantly longer in patients with reported consanguinity vs patients without reported consanguinity. Interestingly they are also significantly longer in patients without reported consanguinity compared with German controls indicating inbreeding in the Arab communities (*p* = 0.0053). **b** ROHs in patients with “unsolved” exomes compared with patients with solved/likely solved exomes. ROHs were significantly longer in solved/likely solved cases suggesting that the higher ROHs result in a better chance to find the causative variant. **c** total number and percentage of families with pathogenic/likely pathogenic variants, candidate variants, and unsolved families.

Statistical analysis was performed using JMP 14.2.0. The nonparametric comparison in Fig. 1 was done using the Wilcoxon method.

## Results

Eighty-three Arab families with at least two affected patients with similar neurological disorders were included in this study. Sixty families reported consanguineous marriages. Twenty-three families were not aware of any consanguinity in their family history. In total 102 individuals received exome sequencing. On average 116,282,290 sequence reads of 101-bp length were generated per sample. The mean sequencing depth was 114× while 92% of the target sequence was covered at least 20 times. Our standardized exome filtering approach revealed pathogenic/likely pathogenic variants in well-established disease genes in 35 families. Probably disease-causing candidate variants that could not be classified as pathogenic or likely pathogenic due to ACMG standards were identified in five families. In addition, two new disease genes (published separately) were identified in the context of this study [18, 19]. In total, 51% of the families (42 families) received either a definitive diagnosis (37 families, Table 1) or a likely diagnosis with a novel candidate variant (5 families, Table 2). Two independent genetic disorders were identified in one family (GPT2 and likely autosomal recessive deafness) as discussed elsewhere [20]. Based on exome sequencing (ROH) were determined for every sequenced patient. The total length of ROHs was used to estimate the degree of inbreeding. patients from families with reported consanguinity had a median ROHs length of 243 Mb. ROHs in these families were significantly longer compared with patients without reported consanguinity of the parents (median ROHs length 48 Mb; *p* < 0.0001) (Fig. 1). Compared with a German control cohort (median ROHs length 35 Mb) the ROHs were significantly longer not only in the Arab patient group with reported consanguinity but also in the Arab patient group without reported consanguinity. A considerably higher percentage of families with reported consanguinity received a genetic diagnosis and the ROHs were also significantly longer in patients with identified pathogenic/likely pathogenic variants compared with patients that did not receive a genetic diagnosis (median ROH in solved patients: 233 Mb, median ROH in patients without genetic diagnosis: 101 Mb; *p* < 0.0001, Fig. 1).

**Table 2:** Overview of identified new candidate variants in established disease genes.

Gene	MIM	Affected/unaffected	FamilyID	Phenotype	Variant	Zygosity	Inheritance
MCOLN1	252650	6/11	TR14	Cerebellar ataxia, affection of upper motor neuron, intellectual disability, epilepsy, pes cavus	NM_020533.3:c.230C>T: p.(Thr77Met)	hom	AR
MFN2	617087	2/3	TR44A	Peripheral motor neuropathy	NM_014874.3:c.1963A>G: p.(Lys655Glu)	hom	AR
*SEPSECS*	613811	2/3	MA01	Intellectual disability, pontocerebellar hypoplasia	NM_016955.4:c.181A>G: p.(Met61Val)	hom	AR
*SYNJ1*	617389	2/3	BE09	Epileptic encephalopathy, severe myoclonic epilepsy	NM_203446.2:c.1274G>T: p.(Cys425Phe)	hom	AR
*TSEN54*	277470	2/3	TR39	Intellectual disability, microcephalus, hypotonia	NM_207346.3:c.341C>T: p.(Pro114Leu)	hom	AR

Detailed annotations can be found in Table S1.

*AR* autosomal recessive, *MIM* mendelian inheritance in men.

As expected most identified variants were homozygous variants in recessive disease genes (Table 1), all located within a region of homozygosity. Autosomal dominant pathogenic variants were identified in two families in the *KCNQ2 gene* and the *KIF11 gene*, respectively. One family had a hemizygous pathogenic variant in *MECP2*. Compound heterozygous pathogenic variants, as well as likely pathogenic copy number variants, were not identified. Of the 37 variants that were regarded as disease causing (in 42 families), 20 variants had been previously associated with disease, 2 variants were established in new disease genes, while 16 were novel variants in known disease genes (6 missense variants, 10 loss of function variants), thus expanding the genetic spectrum of these disorders (Table 1). Of the novel missense variants, only one missense variant in *NTRK1* could be classified as likely pathogenic, due to the pathognomonic phenotype with insensitivity to pain and anhidrosis. The other five missense variants are thus listed below as novel candidate variants.

### Novel candidate variants in established disease genes

Based on the ACMG criteria most novel missense variants cannot be classified as likely pathogenic or pathogenic, if they are found only in one family and no functional readout is available, even if other missense variants in patients with similar phenotypic features have been established in these disease genes. We still consider the following candidate variants as probably pathogenic:

1. *MCOLN1* (homozygous c.230C>T: p.(Thr77Met)): this variant segregated in a large consanguineous family with 6 affected and 11 healthy family members was very rare in gnomAD (MAF 2 × 10^−5^, not observed homozygous) and had high in silico prediction scores (CADD 18.8). The affected patients suffered from mild intellectual disability, slowly progressive cerebellar ataxia, and variable affection of upper motor neuron, epilepsy, and pes cavus. Ophthalmological examination was not performed and corneal clouding was not obvious. Although not being the typical presentation of mucolipidosis type 4 (intellectual disability and ophthalmological abnormalities), the clinical features are compatible with this diagnosis.
2. *MFN2* (homozygous c.1963A>G: p.(Lys655Glu)): this *MFN2* variant segregated in a family with two affected and three healthy family members. Both patients suffered from peripheral neuropathy with distal muscle weakness, suggestive for Charcot–Marie–Tooth Disease. We thus consider CMT2A2B to be the likely diagnosis.
3. *SEPSECS* (homozygous c.181A>G: p.(Met61Val)): this variant was identified in a family with two siblings suffering from pontocerebellar hypoplasia and severe global developmental delay. The variant was very rare in gnomAD (MAF 4 × 10^−6^, not observed homozygous) and received a high in silico prediction score (CADD: 17). We concluded that pontocerebellar hypoplasia type 2D is the likely diagnosis.
4. *SYNJ1* (homozygous c.1274G>T: p.(Cys425Phe)): this variant was present in a family with two affected children suffering from a severe early-onset epileptic encephalopathy with myoclonic epilepsy. The variant was not present in gnomAD, received high in silico prediction scores (CADD: 27.6), and segregated in the family with the parents and one unaffected sibling. We consider this variant to be another cause of autosomal recessive SYNJ1-associated epileptic encephalopathy. So far less than ten patients have been described [21–23].
5. *TSEN54* (homozygous c.341C>T: p.(Pro114Leu)): two siblings with early-onset epilepsy, global developmental delay, and microcephaly shared this variant. The variant was absent from gnomAD, received high in silico prediction scores (CADD: 24.3), and segregated in this family with three healthy family members. Although a brain MRI was not available the clinical features are suspicious for TSEN54 associated pontocerebellar ataxia.

### Extensions of the phenotypic spectrum and novel disease genes

**Fig. 2:**
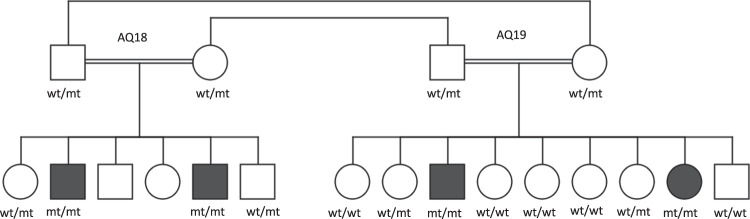
Pedigree of the two related consanguineous families AQ18 and AQ19. The mother in AQ18 is the sister of the father in AQ19 while the mother in AQ19 is the sister of the father in AQ18. In addition, both couples are first cousins. The MTMR2 variant (NM_016156:exon8:c.766_767del) was homozygous in all affected patients and heterozygous in the parents. mt pathogenic variant; wt wild type.

Two distally related families were included in this study with two sibs in each branch suffering from early-onset generalized muscle weakness. Spinal muscular atrophy was suspected due to severe muscle weakness, atrophy, and fasciculations of the tongue in all patients. Additional diagnostics like electrophysiology were not available to these patients from the Palestinian Authority. WES was carried out in one affected individual of both families and revealed a novel homozygous *MTMR2* frameshift variant c.766_767delAA segregating with the disease in both families (Fig. 2). We reexamined all patients clinically to confirm the diagnosis of severe Charcot–Marie–Tooth type 4b1, caused by recessive pathogenic variants in *MTMR2*. The four patients were between 7 and 23 years old. All shared distally pronounced symmetric flaccid weakness, more severe in the older patients. Reflexes were reduced (in the 7-year-old girl) or absent (in all other patients). While the 7-year-old girl was still able to walk independently, the 15-year-old patient was using a walker, and the two oldest patients were wheelchair bound since the age of 14 and 15 years, respectively. All had severe respiratory problems with stridor and breathing restricted to the diaphragm in the two oldest patients. Three of the four patients presented with hoarseness. Facial weakness, chewing, and swallowing difficulties were present in all four patients. Only some minor distal sensory deficits were reported by the patients, and there were only minor deficits in position sense at the toes. Taken together the core features of early-onset disease with respiratory distress, distal symmetric weakness and atrophy, and vocal cord involvement were in agreement with the genetic diagnosis of CMT type 4B1. The early involvement of the vocal cord and stridor is increasingly recognized in patients with CMT type 4B1 [24]. This example shows the importance of reverse phenotyping in these cases, once a genetic diagnosis was suspected.

In fact the a priori clinical diagnosis of the referring local physicians differed several times from the diagnosis achieved after genetically guided reverse phenotyping. Another example for this was a family in which we identified a known pathogenic homozygous *SPG11* variant via WES (AQ54, Table 1), whereas the a prior diagnosis of this family was muscular dystrophy. The more detailed genetically guided phenotyping finally confirmed a complicated form of hereditary spastic paraplegia (cHSP) compatible with the homozygous pathogenic variant in the *SPG11* gene.

Another interesting finding was a homozygous frameshift variant (c.446delG: p.(Ser149Thrfs*45)) in SCL25A15 in two sisters presenting with spastic paraparesis, mild cerebellar ataxia, and polyneuropathy, best summarized as complicated form of HSP. Although pyramidal and cerebellar affections are well-described in patients with recessive pathogenic *SLC25A15* variants [25, 26], hyperornithinemia–hyperammonemia–homocitrullinemia (HHH) syndrome is most likely not on the list when seeing patients clinically presenting with cHSP. An obvious learning disability was not present in the patient’s medical history or clinical impression.

Besides new variants in established disease genes, the WES-first approach enabled us to identify new disease genes for ultra-rare disorders. In families without likely disease-causing variants in the index patient we performed additional WES of a second or third affected family member. This helped to reduce the number of candidate genes and helped (i) to identify two novel disease genes (WWOX and *PAX7*, both published elsewhere [18, 19]) and (ii) to establish substantial expansions of the gene-associated phenotypic spectrum like in *GPT2*, *CNTNAP1*, and *POLR3A* [20, 27, 28]. (iii) We identified additional families with pathogenic or likely pathogenic variants in genes that had been described only in single families. This helped to confirm the pathogenic role of these variants and to establish its causal relation for the disease like the homozygous splice variant c.2023-2A>G in *SCAPER* that has recently been associated with retinitis pigmentosa and intellectual disability [29]. Interestingly, all three patients with this pathogenic *SCAPER* variant in our series had nuclear cataracts in addition to retinitis pigmentosa and intellectual disability.

### WES enables specific therapy

In 4 of 83 families we identified genetic diseases that offer causal treatment options most likely with beneficial effects on the course of disease. The above mentioned molecular genetic diagnosis of HHH syndrome, caused by an defect of the urea cycle, enabled a dietary treatment with supplementation of ornithine, and restriction of protein [25].

Similarly, in three apparently independent families we found a well-established pathogenic *GAMT* variant causing cerebral creatine deficiency syndrome 2 (CCDS2). In CCDS2 standardized treatment recommendations, including creatine supplementation to reduce cerebral creatinine deficiency are available and are likely to improve or stabilize symptoms. Unfortunately, the treatment response could only be monitored in two patients with CCDS2. These two patients both showed improvement of aggressive behavior and autistic features, as well as a reduction of seizures frequency comparable to the previously reported positive effects of creatine supplementation [30]. In summary, WES enabled specific treatment in nine patients from four families.

## Discussion

We have shown that first-line exome diagnostic in neurological patients from consanguineous Arab communities in Israel and the Palestinian Authority reaches a high diagnostic yield. In this study 42 of 83 (51%) families received a definite genetic diagnosis or at least a very likely candidate variant. This is comparable to the diagnostic yield from similar studies using next-generation sequencing in consanguineous populations (55–60%) [31–33].

ROHs were significantly longer in the included Arab patients compared with a German control cohort, even in the subgroup of Arab patients without reported consanguinity. This confirms the basic assumption that there is generally a higher inbreeding rate in the Palestinian and Israeli Arab communities than in other populations even if consanguinity is not documented in the family, and demonstrates the potential of ROHs calculations based on NGS data as a marker for inbreeding. Furthermore, a correlation between longer ROHs and the likelihood of finding a homozygous disease-causing variant was shown.

In our exome-first approach we could establish a genetic diagnosis even with only basic clinical information and without extensive additional diagnostics like electrophysiology, laboratory screening, or brain imaging. For patients from a consanguineous background with limited access to medical diagnostics, first-line WES in combination with careful reverse clinical phenotyping might be the fastest and the most cost-efficient way to establish a genetic diagnosis.

## Supplementary information

supplementary Table 1

## Data Availability

Human variants and phenotypes have been reported to ClinVar (submission name “TLP001,” accession numbers for all variants are found in Table S1; www.ncbi.nlm.nih.gov/clinvar).
