# Correction to: First-line exome sequencing in Palestinian and Israeli Arabs with neurological disorders is efficient and facilitates disease gene discovery

**DOI:** 10.1038/s41431-021-00909-7

**Published:** 2021-05-28

**Authors:** Holger Hengel, Rebecca Buchert, Marc Sturm, Tobias B. Haack, Yvonne Schelling, Muhammad Mahajnah, Rajech Sharkia, Abdussalam Azem, Ghassan Balousha, Zaid Ghanem, Mohammed Falana, Osama Balousha, Suhail Ayesh, Reinhard Keimer, Werner Deigendesch, Jimmy Zaidan, Hiyam Marzouqa, Peter Bauer, Ludger Schöls

**Affiliations:** 1 Department of Neurology and Hertie-Institute for Clinical Brain Research, University of Tübingen, Tübingen, Germany; 2 German Center of Neurodegenerative Diseases (DZNE), Tübingen, Germany; 3 Institute of Medical Genetics and Applied Genomics, University of Tübingen, Tübingen, Germany; 4 The Ruth and Bruce Rappaport Faculty of Medicine, Technion, Haifa, Israel; 5 Child Neurology and Development Center, Hillel-Yaffe Medical Center, Hadera, Israel; 6 Unit of Nature Science, Beit-Berl Academic College, Beit-Berl, Israel; 7 Unit of Human Biology and Genetics, The Triangle Regional Research and Development Center, Kafr Qari, Israel; 8 Department of Biochemistry and Molecular Biology, Faculty of Life Sciences, Tel-Aviv University, Tel-Aviv, Israel; 9 Department of Pathology and Histology, Al-Quds University, Eastern Jerusalem, Palestine; 10 Palestine Medical Complex, Ramallah, Palestine; 11 Faculty of Medicine, Al-Quds University, Eastern Jerusalem, Palestine; 12 Molecular Genetics Laboratory, Al-Makassed Islamic Charitable Hospital, Jerusalem, Israel; 13 Caritas Baby Hospital Bethlehem, Bethlehem, Palestine; 14 Centogene AG (Rostock), Tübingen, Germany

**Keywords:** Genetics research, Neurological disorders, Genetic testing

Correction to: *European Journal of Human Genetics*

10.1038/s41431-020-0609-9

The article “First-line exome sequencing in Palestinian and Israeli Arabs with neurological disorders is efficient and facilitates disease gene discovery,” written by Holger Hengel et al., was originally published electronically on the publisher’s internet portal on 25 March 2020 without open access. With the author’ decision to opt for Open Choice the copyright of the article changed on 17 May 2021 to © Author(s) 2020 and the article is forthwith distributed under a Creative Commons Attribution 4.0 International License, which permits use, sharing, adaptation, distribution, and reproduction in any medium or format, as long as you give appropriate credit to the original author(s) and the source, provide a link to the Creative Commons licence, and indicate if changes were made. The images or other third party material in this article are included in the article’s Creative Commons licence, unless indicated otherwise in a credit line to the material. If material is not included in the article’s Creative Commons licence and your intended use is not permitted by statutory regulation or exceeds the permitted use, you will need to obtain permission directly from the copyright holder. To view a copy of this licence, visit http://creativecommons.org/licenses/by/4.0.

Open access funding enabled and organized by Projekt DEAL.

The original article has been corrected.

